# Increased Spontaneous Central Bleeding and Cognition Impairment in APP/PS1 Mice with Poorly Controlled Diabetes Mellitus

**DOI:** 10.1007/s12035-015-9311-2

**Published:** 2015-07-09

**Authors:** Juan José Ramos-Rodriguez, Carmen Infante-Garcia, Lucia Galindo-Gonzalez, Yaiza Garcia-Molina, Alfonso Lechuga-Sancho, Mónica Garcia-Alloza

**Affiliations:** Division of Physiology, School of Medicine, Instituto de Biomoleculas (INBIO), Universidad de Cadiz, Plaza Fragela 9, 4 piso, 410, Cadiz, 11003 Spain; Department of Radiology and Pediatrics, Universidad de Cadiz, Cadiz, 11003 Spain

**Keywords:** Alzheimer’s disease, Vascular dementia, Diabetes, Streptozotocin, Hemorrhage, Amyloid-beta

## Abstract

Alzheimer’s disease (AD) and vascular dementia (VaD) are the most common causes of dementia, and borderlines are blurred in many cases. Aging remains the main risk factor to suffer dementia; however, epidemiological studies reveal that diabetes may also predispose to suffer AD. In order to further study this relationship, we have induced hypoinsulinemic diabetes to APPswe/PS1dE9 (APP/PS1) mice, a classical model of AD. APP/PS1 mice received streptozotocin (STZ) ip at 18 weeks of age, when AD pathology is not yet established in this animal model. Cognition was evaluated at 26 weeks of age in the Morris water maze and the new object discrimination tests. We observed that STZ-induced episodic and working memory impairment was significantly worsened in APP/PS1 mice. Postmortem assessment included brain atrophy, amyloid-beta and tau pathology, spontaneous bleeding, and increased central inflammation. Interestingly, in APP/PS1-STZ diabetic mice, we detected a shift in Aβ soluble/insoluble levels, towards more toxic soluble species. Phospho-tau levels were also increased in APP/PS1-STZ mice, accompanied by an exacerbated inflammatory process, both in the close proximity to senile plaque (SP) and in SP-free areas. The presence of hemorrhages was significantly higher in APP/PS1-STZ mice, and although pericytes and endothelium were only partially affected, it remains possible that blood-brain barrier alterations underlie observed pathological features. Our data support the implication of the diabetic process in AD and VaD, and it is feasible that improving metabolic control could delay observed central pathology.

## Background

Alzheimer’s disease (AD) and vascular dementia (VaD) are the first and second most common causes of dementia respectively. Moreover, the borderlines between them are blurred, since in many cases, traditional AD hallmarks coexist with markers of vascular injury [1]. AD pathological features include senile plaque (SP), composed by amyloid-beta (Aβ) 40 and 42, neurofibrillary tangles formed by abnormally phosphorylated tau, and synaptic and neuronal loss [2]. On the other hand, VaD is a heterogeneous disease that includes microinfarcts, small vessel ischemic disease, or microvascular injury [1].

Aging remains the main risk factor to suffer AD however, the fact that most of the cases are sporadic has favored the study of other risk factors, among which diabetes plays a relevant role. Impaired insulin secretion and resistance, as well as glucose intolerance, seem to be associated with increased risk of AD [3–5]. Insulin and insulin receptors are widely distributed in regions implicated in learning and memory, such as the cortex and hippocampus, and previous studies have reported cognition impairment in type 1 diabetic children [6, 7]. Also, AD brains present lower insulin levels and higher insulin receptor density when compared to control patients (for a review, see [8]). It has also been shown that insulin dysregulation may interfere with AD neuropathological features, and insulin exacerbation of inflammatory responses may interact with Aβ processing and deposition (for review, see [9, 10]). Tau phosphorylation is also severely affected when insulin levels are altered [11–13], and sequential phosphorylated residues have been characterized when insulin levels are reduced [14]. Insulin participates in neurovascular control, and therefore, it may relate metabolic alterations to VaD [15]. Also, vascular damage has been suggested to reduce Aβ clearance along interstitial fluid drainage pathways [16, 17] linking diabetes, AD and VaD. Following this idea, the beneficial effect of intranasal insulin on cognitive function in AD patients has been reported [18, 19]. In order to further explore the relationship between metabolic alterations, as insulin deprivation and derived hyperglycemia, and AD-VaD central pathology, we have induced type 1 diabetes (T1D) to a classical model of AD. We injected STZ to APP/PS1mice before Aβ deposition commences, at around 4 months of age, and we have observed a significant worsening effect of cognitive abilities, including episodic memory, by 6 months of age, when Aβ deposition is established in APP/PS1 mice [20]. As previously described, tau phosphorylation was significantly increased, both in the cortex and hippocampus from APP/PS1-STZ mice. Interestingly, a shift in Aβ soluble/insoluble levels was observed, with increased soluble, more toxic, species found in APP/PS1-STZ mice, accompanied by reduced insoluble Aβ levels and deposited amyloid. Also, inflammation was exacerbated, and increased microglial activation was observed in APP/PS1-STZ mice. Moreover, we observed that hemorrhage burden was worsened in APP/PS1-STZ mice, in relevant regions for learning and memory, suggesting that alterations in the blood-brain barrier may be responsible for the pathological features detected in APP/PS-STZ mice.

## Results

### Metabolic Characterization

No differences were detected in any of the parameters under study before administering STZ (18 weeks) (body weight *F*_(3,33)_ = 0.691, *p* = 0.564, glucose *F*_(3,33)_ = 1.49, *p* = 0.234, and insulin *F*_(3,33)_ = 0.773, *p* = 0.518) (Table 1). As it could be expected, 8 weeks after STZ treatment, we observed that metabolic parameters were significantly altered, resembling T1D with poor metabolic control. A slight reduction in body weight was observed in STZ-treated mice, although differences did not reach statistical significance (*F*_(3,33)_ = 0.691, *p* = 0.564). Glucose levels were significantly increased in STZ-treated mice and this effect was worsened in APP/PS1-STZ mice (*F*_(3,28)_ = 60.26, **p < 0.01 vs. the rest of the groups, **p < 0.01 vs. Wt-Sham and APP/PS1-Sham) (Table 1). Insulin levels were significantly reduced in STZ-treated mice, both Wt and APP/PS1 groups (*F*_(3,28)_ = 54.59, ††*p* < 0.01 vs. Wt-Sham and APP/PS1-Sham) (Table 1).

**Table 1:** Metabolic characterization of STZ-treated mice

	Body weight (g)		Glucose (mg/dl)		Insulin (μg/dl)	
	18 weeks (before STZ)	26 weeks (after STZ	18 weeks (before STZ)	26 weeks (after STZ)	18 weeks (before STZ)	26 weeks (after STZ)
Wt-Sham	27.96 ± 2.18	27.98 ± 2.03	149.99 ± 4.78	150.33 ± 4.78	0.63 ± 0.04	0.57 ± 0.01
Wt-STZ	29.27 ± 1.81	23.31 ± 2.06	135.22 ± 8.54	450.33 ± 41.95††	0.65 ± 0.04	0.23 ± 0.03††
APP/PS1-Sham	25.75 ± 1.98	25.08 ± 2.24	125.25 ± 10.02	123.57 ± 5.40	0.60 ± 0.02	0.56 ± 0.02
APP/PS1-STZ	28.50 ± 1.35	23.46 ± 1.37	125.50 ± 6.97	562.10 ± 21.10**	0.67 ± 0.03	0.23 ± 0.02††

No differences were detected in any of the parameters under study before administering STZ (18 weeks): body weight (*p* = 0.564), glucose (*p* = 0.234), and insulin (p = 0.518). Metabolic parameters were significantly affected at 26 weeks; after STZ treatment, glucose was increased in Wt-STZ mice, and even higher increases were observed in APP/PS1-STZ mice (**p < 0.01 vs. the rest of the groups, ††*p* < 0.01 vs. Wt-Sham and APP/PS1-Sham), insulin was significantly reduced in STZ-treated mice (††*p* < 0.01 vs. Wt-Sham and APP/PS1-Sham), and whereas body weight was slightly reduced in STZ-treated mice, differences did not reach statistical significance (*p* = 0.35). Data are representative of 6–11 mice and differences were detected by one-way ANOVA for independent samples followed by Tukey *b* or Tamhane tests as required

### Learning and Memory Assessment

**Fig. 1:**
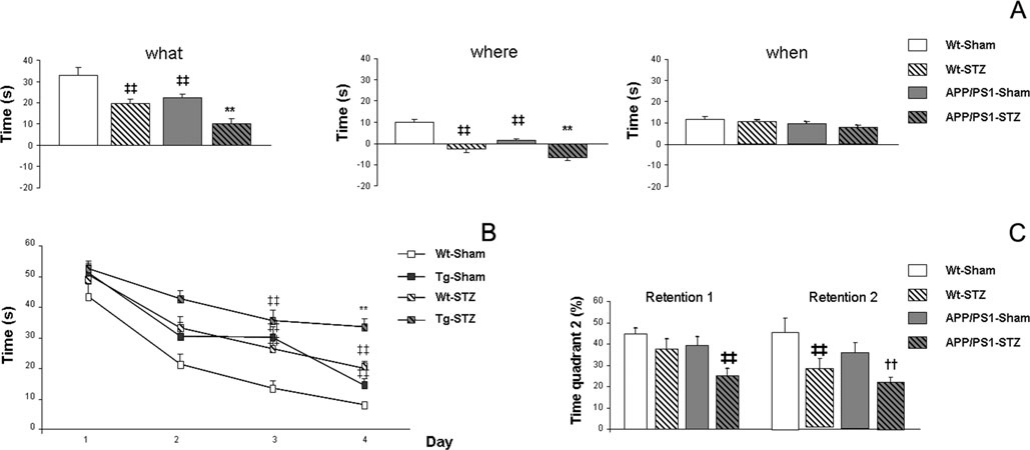
Learning and memory activities are impaired in APP/PS1-STZ mice. **a** The NOD test revealed that episodic memory was compromised in APP/PS1-Sham and Wt-STZ mice and this effect was worsened in APP/PS1-STZ mice for “what” (**p < 0.01 vs. the rest of the groups, ‡‡*p* < 0.01 vs. Wt-Sham) and “where” (**p < 0.01 vs. the rest of the groups, ‡‡*p* < 0.01 vs. Wt-Sham), whereas differences did not reach statistical significance in the case of the “when” paradigm (*p* = 0.251). **b** Spatial learning and memory were also affected in the MWM test, and a significant group × day effect was detected by two-way ANOVA for independent samples (*p = 0.04). Further daily assessment revealed that learning was significantly compromised along the acquisition phase in APP/PS1-Sham and Wt-STZ mice, and this effect was worsened in APP/PS1-STZ mice (day 1 *p* = 0.177, day 2 ‡‡*p* < 0.01 vs. Wt-Sham, day 3 ‡‡*p* < 0.01 vs. Wt-Sham, and day 4 **p < 0.01 vs. the rest of the groups, ‡‡*p* < 0.01 vs. Wt-Sham). **c** A significant impairment was also observed in APP/PS1-STZ mice on the retention 1 (24 h after acquisition) (‡‡*p* < 0.01 vs. Wt-Sham). On retention 2 (72 h after acquisition), we observed an overall worse performance in all groups; Wt-STZ mice were significantly impaired and this effect was exacerbated in APP/PS1-STZ mice (††*p* < 0.01 vs. Wt-Sham and APP/PS1-Sham, ‡‡*p* < 0.01 vs. Wt-Sham). Data are representative of 6–9 mice and differences were detected by one-way ANOVA followed by Tukey *b* or Tamhane tests as required

In order to evaluate episodic memory, we performed the new object discrimination (NOD) test as previously described [21]. To our knowledge, episodic memory in a mixed model of T1D-AD has not been assessed before. We observed an overall cognitive decline in wild-type mice treated with STZ, with comparable performance to APP/PS1-Sham treated mice, and a worsening effect was observed in APP/PS1-STZ mice. Whereas no differences were observed among groups in the “when” paradigm (*F*_(3,83)_ = 1.39, *p* = 0.251), a slight impairment was observed in Wt-STZ, APP/PS1-Sham, and APP/PS1-STZ groups, when compared with Wt-Sham animals (*F*_(3,83)_ = 1.39, *p* = 0.251). On the other hand, a significant impairment was observed in the “what” paradigm, in the case of APP/PS1-Sham and Wt-STZ animals, and a worsening effect was observed in APP/PS1-STZ-treated mice (*F*_(3,83)_ = 15.18, **p < 0.01 vs. the rest of the groups, ‡‡*p* < 0.01 vs. Wt-Sham) (Fig. 1a). A similar profile was observed for the “where” paradigm (*F*_(3,83)_ = 27.34, **p < 0.01 vs. the rest of the groups, ‡‡*p* < 0.01 vs. Wt-Sham) (Fig. 1a).

We also evaluated spatial memory in the Morris water maze (MWM) test, and we detected a significant group × day effect along the acquisition phase, by two-way ANOVA (*F*_(9,465)_ = 1.92, *p = 0.04). Further assessment of individual days revealed an overall increase in the required time to locate the platform for Wt-STZ and APP/PS1-Sham mice, and a worsening effect in APP/PS1-STZ mice, that was especially relevant during the last day of the acquisition phase (day 1 *F*_(3,124)_ = 1.667, *p* = 0.177, day 2 *F*_(3,124)_ = 7.24, ‡‡*p* < 0.01 vs. Wt-Sham, day 3 *F*_(3,124)_ = 9.48, ‡‡*p* < 0.01 vs. Wt-Sham, day 4 *F*_(3,121)_ = 21.43, **p < 0.01 vs. the rest of the groups, ‡‡*p* < 0.01 vs. Wt-Sham] (Fig. 1b). Similar memory impairment was observed during the retention phase. In retention 1 (24 h after completing the acquisition phase), we observed a reduction in the time that APP/PS1-STZ mice spent in the quadrant (quadrant 2) where the platform used to be located (*F*_(3,26)_ = 4,93 ‡‡*p* = 0.008 vs. Wt-Sham) (Fig. 1c). On the second retention phase (72 h after completion of the acquisition phase), we observed that memory dysfunction was statistically significant in the Wt-STZ group, and cognitive impairment was worsened in APP/PS1-STZ animals (*F*_(3,24)_ = 45.13, ††*p* = 0.006 vs. Wt-Sham and APP/PS1-Sham, ‡‡*p* < 0.01 vs. Wt-Sham) (Fig. 1c).

We analyzed motor activity in all animals under study by measuring locomotor activity in the open field, and no differences among groups were observed when we compared the distances in the proximity of the walls (*F*_(3,25)_ = 1.34, *p* = 0.283) or in the center of the arena, indicative of anxiety-like behavior (*F*_(3,25)_ = 1.33, *p* = 0.285) (Table 2). Swimming speed in the MWM also remained unaltered (*F*_(3,26)_ = 0.822, *p* = 0.494). Similarly, final speed in the rotarod test was not affected in any of the groups under study (*F*_(3,24)_ = 1.75, *p* = 0.183). Motor activity or anxiety-related paradigms were unaffected (Table 2), supporting that our observations in learning and memory were not due to motor function limitations. Altogether, our data suggest the presence of cognitive impairment both in AD (APP/PS1-Sham) and diabetic (Wt-STZ) mice, with a synergistic effect in APP/PS1-STZ mice, supporting a cross-talk between both pathology types that worsens episodic and spatial memory.

**Table 2:** No differences in motor function were observed among groups

	Distance of outer arena (cm)	Distance of inner arena (cm)	Swimming speed (cm/s)	Rotarod final speed (rpm)
Wt-Sham	4389.88 ± 612.29	702.47 ± 57.64	13.02 ± 0.82	23.17 ± 2.15
Wt-STZ	3628.93 ± 245.72	646.58 ± 48.45	11.48 ± 0.93	17.67 ± 1.69
APP/PS1-Sham	4709.50 ± 510.19	783.09 ± 63.64	11.19 ± 0.58	16.00 ± 2.82
APP/PS1-STZ	4693.93 ± 373.25	759.09 ± 42.62	11.74 ± 0.82	16.67 ± 2.29

No differences were observed in any of the parameters under study (distance of outer arena, *p* = 0.283; distance of inner arena, *p* = 0.285; swimming speed, *p* = 0.494; rotarod final speed, *p* = 0.183)

### Brain Morphology

**Fig. 2:**
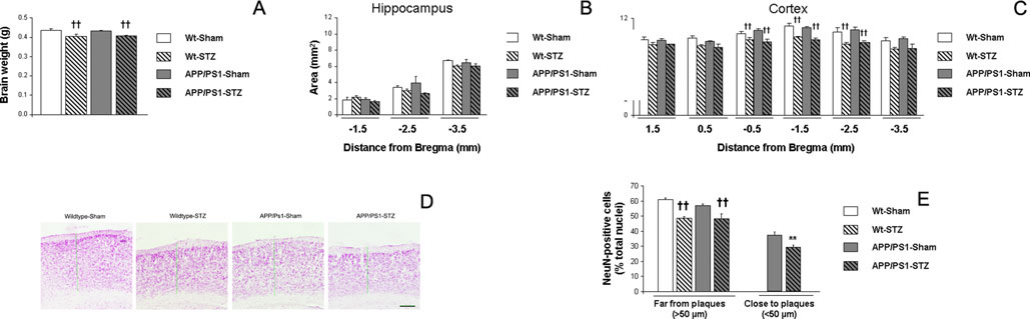
Significant brain atrophy is observed after STZ administration. **a** We observed a reduction of brain weight in Wt-STZ and APP/PS1-STZ-treated mice, at 26 weeks of age (††*p* < 0.01 vs. Wt-Sham and APP/PS1-Sham). **b** Hippocampal assessment revealed a slight reduction in APP/PS1-STZ-treated mice, although differences did not reach statistical significance (−1.5 mm, *p* = 0.526; −2.5 mm, *p* = 0.303; −3.5 mm, *p* = 0.289). **c** Further assessment of cortical brain sections revealed an overall reduction of cortex size after STZ treatment, that was slightly worsened in APP/PS1 mice. Cortical size reduction reached statistical significance at −0.5, −1.5, and −2.5 mm from bregma (††*p* < 0.01 vs. wild type and APP/PS1). **d** Illustrative images of cortical size and thickness, stained with cresyl violet, in all groups under study, where significant shrinkage can be observed in STZ-treated mice. *Green lines* point at cortical thickness (Wt-Sham = 1.006 mm, Wt-STZ = 0.887 mm, APP/PS1-Sham = 0.96 mm, and APP/PS1-STZ = 0.825 mm). *Scale bar* = 250 μm. **e** A significant reduction in the proportion of neurons was observed in wild-type-STZ mice far from plaques, and this effect was worsened in APP/PS1-STZ mice (*F*_(3,596)_ = 11.27, ††*p* < 0.01 vs. Wt-Sham and APP/PS1-Sham). An overall reduction in proportion of NeuN-positive cells was observed in the proximity to senile plaques, and this effect was more severe in APP/PS1-STZ-treated mice (**p = 0.001 vs. APP/PS1 Sham)

Brain weight was significantly reduced in STZ-treated mice and APP/PS1-STZ mice were more severely affected, although no differences were detected when compared with wild-type STZ-treated animals (*F*_(3,26)_ = 7.18, ††*p* < 0.01 vs. Wt-Sham and APP/PS1-Sham) (Fig. 2a). Since brain weight is a very rough approximation to measure brain atrophy, cresyl violet staining was performed on brain hemisections and cortical and hippocampal sizes were measured along the hemisphere. We detected that hippocampal areas were slightly reduced in STZ-treated mice, although no statistical differences were observed (−1.5 mm [*F*_(3,13)_ = 0.780, *p* = 0.526], −2.5 mm [*F*_(3,14)_ = 1.33, *p* = 0.303], −3.5 mm [*F*_(3,14)_ = 21.38, *p* = 0.289], at least at this stage of the disease (Fig. 2b)). An overall reduction in cortical size was observed in STZ-treated mice, especially evident in APP/PS1-STZ mice (1.5 mm [*F*_(3,14)_ = 41.55, *p* = 0.244], 0.5 mm [*F*_(3,13)_ = 3.30, *p* = 0.054], −0.5 mm [*F*_(3,13)_ = 7.84, ††*p* = 0.003 vs. Wt-Sham and APP/PS1-Sham], −1.5 mm [*F*_(3,13)_ = 15.24, ††*p* < 0.001 vs. Wt-Sham and APP/PS1-Sham], −2.5 mm [*F*_(3,13)_ = 7.84, †*p* = 0.003 vs. Wt-Sham and APP/PS1-Sham], and −3.5 mm [*F*_(3,14)_ = 2.64, *p* = 0.09]) (Fig. 2c, d).

The specific effect of the atrophy process on neurons was also assessed, and the percentage of neuronal nuclei (NeuN)-positive cells was significantly reduced in STZ-treated mice, both in wild-type and APP/PS1 mice (Fig. 2e). The percentage of NeuN-positive cells was lower in the proximity of SP, and APP/PS1-STZ mice presented even lower densities when compared with APP/PS1-Sham mice (Fig. 2e).

### Amyloid-Beta Pathology

**Fig. 3:**
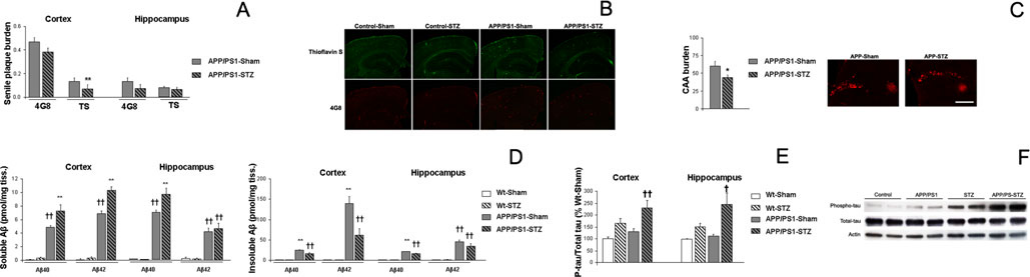
Cortical and hippocampal amyloid pathology after STZ treatment. **a** A slight reduction of cortical SP burden was observed after STZ treatment, although differences only reached statistical significance in the case of TS staining (4G8 *p* = 0.085, TS **p < 0.01 vs. APP/PS1-Sham). A similar profile was observed in the hippocampus although no statistical differences were detected (4G8 *p* = 0.272, TS *p* = 0.577). **b** Illustrative example of TS staining (*green*) and 4G8 (*red*) immunostaining in wild-type-Sham, wild-type-STZ, APP/PS1-Sham, and APP/PS1-STZ mice. *Scale bar* = 200 μm. **c** CAA burden was also slightly lower in APP/PS1-STZ mice (*p = 0.022 vs. APP/PS1). Illustrative images of leptomeningeal vessels with CAA stained with 4G8. *Scale bar* = 100 μm. **d** Soluble Aβ40 and 42 levels were increased both in the cortex and hippocampus from APP/PS1-STZ-treated mice (cortex Aβ40 **p < 0.01 vs. the rest of the groups, ††*p* < 0.01 vs. wild-type-Sham and wild-type-STZ, Aβ42 **p < 0.01 vs. the rest of the groups, ††*p* < 0.01 vs. wild-type-Sham and Wild-type-STZ; hippocampus Aβ40 **p < 0.01 vs. the rest of the groups, ††*p* < 0.01 vs. wild-type-Sham and wild-type-STZ, Aβ42 ††*p* < 0.01 vs. wild-type-Sham and wild-type-STZ). On the other hand, insoluble Aβ40 and 42 levels were reduced in APP/PS1-STZ mice when compared to sham-treated animals both in the cortex (Aβ40, **p < 0.01 vs. the rest of the groups, ††*p* < 0.01 vs. wild-type-Sham and wild-type-STZ; Aβ42, **p < 0.01 vs. the rest of the groups, ††*p* < 0.01 vs. wild-type-Sham and wild-type-STZ) and the hippocampus (Aβ40, **p < 0.01 vs. the rest of the groups, ††*p* < 0.01 vs. wild-type-Sham and wild-type-STZ; Aβ42, ††*p* < 0.01 vs. wild-type-Sham and wild-type-STZ). **e** Phospho-tau/total tau ratio was significantly increased in APP/PS1-STZ-treated mice both in the cortex (††*p* = 0.003 vs. wild-type-Sham and APP/PS1-Sham) and in the hippocampus (†*p* = 0.011 vs. wild-type-Sham and APP/PS1-Sham). **f** Illustrative example of cortical tau phosphorylation in wild-type-Sham, wild-type-STZ, APP/PS1-Sham, and APP/PS1-STZ mice

In order to determine the effect of STZ on Aβ pathology, we quantified SP in the cortex and hippocampus of APP/PS1-Sham and APP/PS1-STZ mice. We randomly stained wild-type sections (from Sham and STZ-treated mice), and no SPs were detected by immunohistochemistry with 4G8 antibody or staining with thioflavin S (TS) (Fig. 3b). SP burden was slightly reduced in the cortex from APP/PS-STZ mice after 4G8 immunohistochemistry, although differences just missed statistical significance (*p* = 0.052) (Fig. 3a, b). As in previous studies [20], the overall SP size was not altered (APP/PS1-Sham 273.84 ± 7.64 μm^2^ and APP/PS1-STZ 267.46 ± 8.11 μm^2^, *p* = 0.571), supporting the idea that SP size is stable once deposited [17, 22]. A slight, non-significant, reduction in the number of SP per square millimeter was detected in APP/PS1-STZ-treated mice (APP/PS1-Sham 17.78 ± 1.23 SP/mm^2^ and APP/PS1-STZ 14.99 ± 1.04 SP/mm^2^, *p* = 0.089). When dense-core SP burden was measured after TS staining, we observed a significant reduction of affected cortical area in APP/PS1-STZ-treated mice (**p < 0.01 vs. APP/PS1-Sham) (Fig. 3a). As expected in this animal model, SP burden was lower in the hippocampus, and although we observed a similar trend to that described in the cortex, differences did not reach statistical significance when SP deposits inmunostained with 4G8 were quantified (*p* = 0.272) (Fig. 3a). Similarly, neither 4G8 deposits size (APP/PS1-Sham 232.58 ± 15.73 μm^2^ and APP/PS1-STZ 253.85 ± 22.62 μm^2^, *p* = 0.432) or number per square millimeter (/PS1-Sham 7.79 ± 1.40 μm^2^ and APP/PS1-STZ 5.29 ± 0.86 μm^2^, p = 0.147) was affected in the hippocampus. TS deposits also reproduced this profile (*p* = 0.577) (Fig. 3a). We observed a similar effect when cerebral amyloid angiopathy (CAA) was quantified, and a reduction of CAA was observed in APP/PS1-STZ mice (*p = 0.022 vs. APP/PS1-Sham) (Fig. 3c). Immunohistochemistry observations were corroborated by Aβ ELISA studies. As expected, Aβ levels were significantly increased in APP/PS1 mice, when compared with wild-type animals, and no differences were observed between wild-type or wild-type-STZ-treated mice. Whereas an increase of soluble Aβ40 [*F*_(3,10)_ = 39.50, **p < 0.01 vs. the rest of the groups, ††*p* < 0.01 vs. wild type and wild-type-STZ] and Aβ42 [*F*_(3,10)_ = 143.81, **p < 0.01 vs. the rest of the groups, ††*p* < 0.01 vs. wild type and wild-type-STZ] levels was observed in the cortex from APP/PS1-STZ-treated mice (Fig. 3D), a reduction of insoluble species was observed in APP/PS1-STZ-treated mice (Aβ40 [*F*_(3,9)_ = 61.34, **p < 0.01 vs. the rest of the groups, ††*p* < 0.01 vs. wild type and wild-type-STZ], Aβ40 [*F*_(3,10)_ = 22.04, **p < 0.01 vs. the rest of the groups, ††*p* < 0.01 vs. wild type and wild-type-STZ]) (Fig. 3d). A similar profile was observed in the hippocampus although differences only reached statistical significance when Aβ40 species were compared, both in the case of soluble (Aβ40 [*F*_(3,10)_ = 81.93, **p < 0.01 vs. the rest of the groups, ††*p* < 0.01 vs. wild type and wild-type-STZ], Aβ42 [*F*_(3,10)_ = 18.513, ††*p* < 0.01 vs. wild type and wild-type-STZ]) (Fig. 3d) and insoluble Aβ levels ([*F*_(3,10)_ = 164.24, **p < 0.01 vs. the rest of the groups, ††*p* < 0.01 vs. wild type and wild-type-STZ], Aβ42 [*F*_(3,10)_ = 21.93, ††*p* < 0.01 vs. wild type and wild-type-STZ]). These data suggest a shift between soluble-insoluble Aβ species in APP/PS1-STZ-treated mice that might be due to alterations in any of the steps implicated in Aβ production or clearance.

### Tau Pathology

We measured the ratio of phospho-tau/total tau levels by Western blot. A slight increase in cortical phospho-tau/total tau ratio was observed in APP/PS1-Sham and Wt-STZ mice, although differences only reached statistical significance in APP/PS1-STZ-treated mice (*F*_(3,26)_ = 5.91, ††*p* = 0.003 vs. Wt-Sham and APP/PS1-Sham) (Fig. 3e, f). A similar profile was observed in the hippocampus (*F*_(3,27)_ = 4.51, †*p* = 0.011 vs. Wt-Sham and APP/PS1-Sham) (Fig. 3e). Our data are in accordance with previous studies where significant alterations, in central tau phosphorylation, are observed after STZ diabetes induction [11, 14, 23].

### IDE and Neprilysin

When we analyzed central levels of enzymes implicated in insulin and Aβ degradation, we did not observe any alterations in the cortex or the hippocampus from any of the groups under study (Table 3). After STZ treatment, insulin degrading enzyme (IDE) levels were unaltered in the cortex (*F*_(3,14)_ = 0.87, *p* = 0.47) or the hippocampus (*F*_(3,14)_ = 0.054, *p* = 0.98), and a similar outcome was observed when neprilysin levels were compared (cortex *F*_(3,14)_ = 10.46, *p* = 0.709, hippocampus *F*_(3,14)_ = 0.54, *p* = 0.659), suggesting that these enzymes are not affected after treatment (Table 3).

**Table 3:** Insulin degrading enzyme (IDE) and neprilysin were not affected by STZ treatment

	Cortex		Hippocampus	
	IDE (% Wt-Sham)	NEP (% Wt-Sham)	IDE (% Wt-Sham)	NEP (% Wt-Sham)
Wt-Sham	100.00 ± 5.45	100.00 ± 8.39	100.00 ± 14.16	100.00 ± 2.96
Wt-STZ	93.60 ± 10.12	99.94 ± 14.00	97.33 ± 19.37	110.12 ± 11.45
APP/PS1-Sham	79.96 ± 17.76	87.55 ± 10.37	90.46 ± 4.62	96.62 ± 11.19
APP/PS1-STZ	106.39 ± 6.93	106.02 ± 10.90	98.60 ± 31.04	109.76 ± 5.57

No differences were detected when IDE and neprilysin levels were analyzed after STZ treatment in the cortex (IDE *p* = 0.47, neprilysin *p* = 0.709) or the hippocampus (IDE *p* = 0.98, neprilysin *p* = 0.659)

### Microglial Activation

**Fig. 4:**
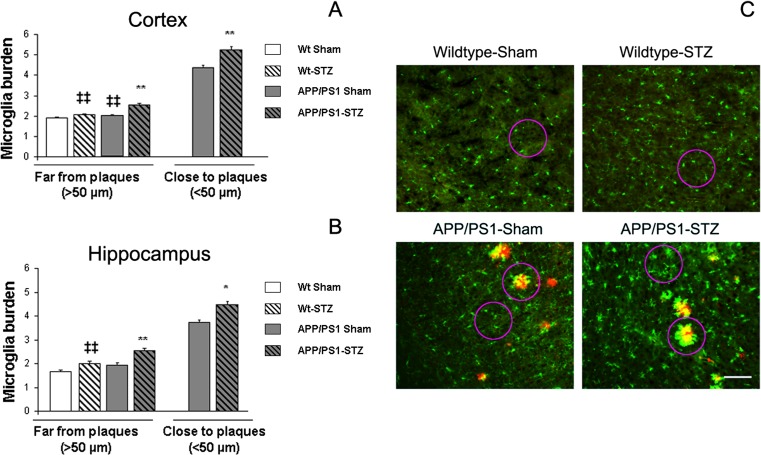
Microglial activation in STZ-treated mice. **a** Microglial burden was significantly increased in SP-free areas in the cortex from APP/PS1-Sham and Wt-STZ-treated mice. Moreover, this effect was worsened in APP/PS1-STZ-treated mice (**p = 0.01 vs. the rest of the groups, ‡‡*p* < 0.01 vs. Wt-Sham). Microglial burden in close proximity to SP was significantly higher in APP/PS1-STZ-treated mice (**p < 0.01 vs. APP/PS1-Sham). **b** A similar profile was observed in the hippocampus, where increased microglial burden in SP-free areas from Wt-STZ-treated mice was worsened in APP/PS1-STZ-treated animals (**p < 0.01 vs. the rest of the groups, ‡‡*p* < 0.01 vs. Wt-Sham). In the proximity of SP, microglial burden was significantly higher in APP/PS1-STZ mice (*p < 0.04 vs. APP/PS1-Sham). **c** Illustrative examples of cortical microglial immunostaining using anti-microglia (iba 1, *green*) and anti-Aβ (4G8, *red*) where increased microglial burden can be detected in APP/PS1-STZ mice, both in proximity to and far from SP. *Scale bar* = 125 μm

We analyzed the inflammatory process by quantifying microglial immunostaining with iba-1 antibody. We observed that microglial burden was slightly increased in the cortex from APP/PS1-Sham mice even far from plaques, and this effect was more pronounced in Wt-STZ-treated mice. Inflammatory process far from plaques was significantly worsened in APP/PS1-STZ-treated mice (*F*_(3,1382)_ = 42.25, **p = 0.01 vs. the rest of the groups, ‡‡*p* < 0.01 vs. Wt-Sham) (Fig. 4a, c), supporting a synergistic effect between diabetes and AD at this level. When we analyzed microglial burden in the close proximity of SP, we also observed that the inflammatory process was favored in APP/PS1-STZ mice (**p < 0.01 vs. APP/PS1-Sham) (Fig. 4a, c). A similar profile was observed in the hippocampus, both far (*F*_(3,272)_ = 42.26.45, **p < 0.01 vs. the rest of the groups, ‡‡*p* < 0.01 vs. Wt-Sham) and close to SP (*p < 0.04 vs. APP/PS1-Sham) (Fig. 4b), supporting an overall increase of microglia in the hippocampus.

### Effect of STZ on Brain Vascular Pathology

**Fig. 5:**
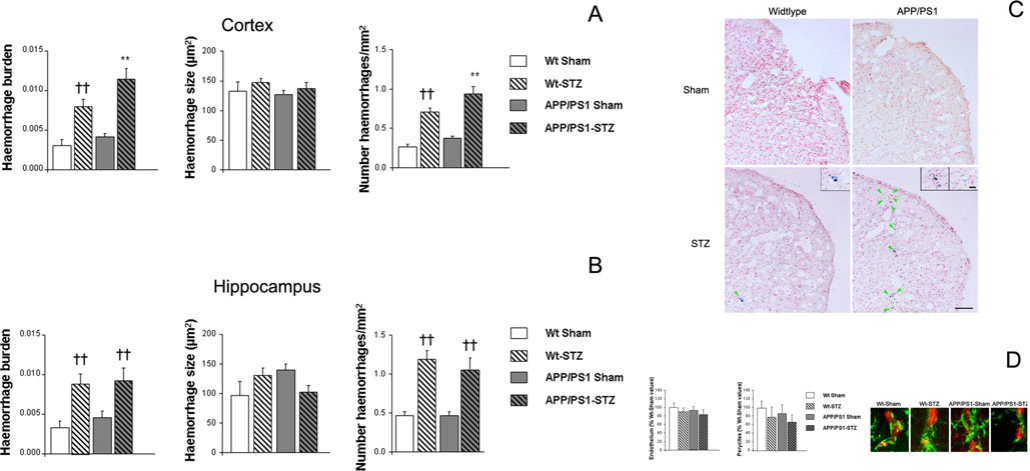
Spontaneous bleeding is significantly increased in APP/PS1-STZ mice. **a** Hemorrhage burden was significantly increased in the cortex from Wt-STZ-treated mice and this effect was worsened in APP/PS1-STZ-treated mice (**p < 0.01 vs. the rest of the groups, ††*p* < 0.01 vs. Wt-Sham and APP/PS1-Sham). Individual hemorrhage size was similar in all groups (*p* = 0.69) under study, whereas a significant increase in the number of hemorrhages per square millimeter was detected in Wt-STZ mice; a worsening effect was observed in APP/PS1-STZ mice (**p < 0.01 vs. the rest of the groups, ††*p* < 0.01 vs. Wt-Sham and APP/PS1-Sham). **b** A similar trend was detected in the hippocampus, and STZ-treated mice presented a significant increase in hemorrhage burden (††*p* < 0.01 vs. Wt-Sham and APP/PS1-Sham), due to an increase in the number of hemorrhages per square millimeter (††*p* < 0.01 vs. Wt-Sham and APP/PS1-Sham), while hemorrhage size was similar in all groups under study (*p* = 0.069). **c** Illustrative example of hemorrhages in cortical sections from all groups under study, where a significant increase in hemorrhage burden can be detected in APP/PS1-STZ mice. *Green arrows* point at hemorrhages stained with Prussian blue and counterstained with neutral red. *Scale bar* = 100 μm. *Insets* show hemorrhagic areas in STZ and APP/PS1-STZ mice, where *light and dark blue spots* can be identified as earlier and older hemorrhages. *Scale bar* = 25 μm. **d** Representative images of endothelium (*red*) and pericyte (*green*) immunostaining in all groups under study. *Scale bar* = 5 μm. We observed an slight, non significant, reduction in the amount of pericytes and endothelium cells in vessels in APP/PS1-STZ mice (endothelium *p* = 0.676, pericytes *p* = 0.521)

We observed an increase of hemorrhage burden in the cortex from STZ-treated mice, and this effect was worsened in APP/PS1 animals (*F*_(3,198)_ = 15.81, **p < 0.01 vs. the rest of the groups, ††*p* < 0.01 vs. Wt-Sham and APP/PS1-Sham). Individual hemorrhage size was similar among groups (*F*_(3,1002)_ = 0.69) although the number of hemorrhages per square millimeter was significantly increased in APP/PS1-STZ mice (*F*_(3,207)_ = 27.25, **p < 0.01 vs. the rest of the groups, ††*p* < 0.01 vs. Wt-sham and APP/PS1-Sham) (Fig. 5a, c). These observations support a synergistic effect between diabetes and AD pathology. Hippocampal studies revealed a similar outcome to a more moderate extent, supporting the preferential affectation of the cortex (hemorrhage burden [*F*_(3,71)_ = 5.61, ††*p* < 0.01 vs. Wt-sham and APP/PS1-Sham], individual hemorrhage size [*F*_(3,260)_ = 0.061], and number of hemorrhages/mm^2^ [*F*_(3,79)_ = 10.09, ††*p* < 0.01 vs. Wt-sham and APP/PS1-Sham]) (Fig. 5b). Endothelial cells and pericyte densities were slightly reduced in APP/PS1-STZ mice, although differences did not reach statistical significance (endothelium [*F*(3,66) = 0.511, *p* = 0.676], pericytes [*F*(3,59) = 0.760, *p* = 0.521]) (Fig. 5c).

## Discussion

Previous clinical and epidemiological studies support the role of metabolic disorders, including midlife hypercholesterolemia or metabolic syndrome, as risk factors to suffer AD [24–27]. It has been reported that insulin plays a relevant role in central nervous system normal activity and that alterations of insulin-dependent functions could be related to central pathological features observed in AD [1, 9, 28]. Following this idea, alterations in insulin levels, such as those observed in T1D [29] and in T2D [3, 5, 30, 31], have been related to AD and VaD. In order to further study this relationship, STZ has been widely used as an alternative model to induce sporadic AD, by central icv administration (for review, see [32]). However, the studies on the relationship between STZ-induced diabetes and AD-VaD alterations have been scarcer.

Our observations in the Morris water maze test are in accordance with previous studies in diabetic models [33, 34]. We detected spatial memory alterations in APP/PS1-Sham mice as well as in Wt-STZ-treated mice, and these effects were worsened in APP/PS1-STZ mice, both during the acquisition and retention phases, suggesting a synergistic effect between diabetes and AD. Previous studies have reported that STZ-induced diabetes may interfere with conditioned fear memory or induce anxiety-like behavior in rodents [35, 36]; however, the fact that we did not observe any motor function alterations or anxiety-related behavior supports that cognitive impairment was not secondary to other central behavioral limitations. In order to further explore cognitive alterations, we explored episodic memory by a demanding approach looking at three aspects of novel object exploration “what,” “where,” and “when.” As far as we know, episodic memory has not been assessed in this animal model before, and we found an overall significant impairment when we analyzed object, place, and temporal order memory in APP/PS1-STZ mice. In our hands, AD develops with greater certainty and severity after insulin deficiency/diabetes gets established. Previous neuropsychological studies in patients have revealed that episodic memory is early affected in the dementia process and that the diabetic process is associated with lower levels of global cognition [37]. Following this idea, general intelligence decline or vocabulary impoverishment has also been observed (for review, see [38]).

STZ treatment induced central atrophy both in APP/PS1 and wild-type mice. Brain weight was reduced and a significant reduction of cortical size was also detected. Similar observations have been previously reported in different models of diabetes [11, 39, 40]. Whereas we did not try to rescue observed abnormalities by insulin supplementation, promising effects have been reported in pilot clinical trials with AD patients [18]. Also, insulin replacement has been shown to successfully recover observed abnormalities in T1D mice [19] or in metabolically impaired AD transgenic mice [41], and therefore, this is a relevant line that should be further explored. Our data are in accordance with studies in T1D patients, where frontal gray matter atrophy or cortical thinning has been reported [42, 43]. It also seems that smaller brain volumes are observed in adults with early onset of diabetes, and since T1D commonly debuts at early age, it is feasible that modifications in regular brain development may also contribute to brain alterations observed in adulthood (for review, see [44]). However, in one-time imaging studies, it is hard to determine which area is earlier or more severely affected, and longitudinal imaging studies in T1D patients would be needed to fully understand the role of T1D in brain atrophy. We further analyzed neuronal loss in STZ mice and an overall reduction in the percentage of NeuN-positive cells was observed. We cannot obviate that STZ is toxic, and therefore, specific central action cannot be excluded; however, the synergistic effect observed in the close proximity to SP in APP/PS1-STZ mice supports a cross-talk between AD and diabetes, as previously described by other groups with similar experimental approaches (for review, see [45]).

When we analyzed AD-related central pathology, we observed a dramatic increase in the phospho-tau/total tau ratio in APP/PS1-STZ-treated mice, both in the cortex and the hippocampus. Studies on 3xTg-AD mice have shown no effect on tau phosphorylation after STZ administration [23]; however, other groups have reported similar findings to ours, in APP/PS1 mice [46], and also increased tau phosphorylation has been observed in different diabetic models (for review, see [10]). Moreover Clodfelder-Miller et al. have sequentially characterized affected phosphorylated tau residues after insulin depletion induced by STZ [14]. They also showed that the process leading to increased tau phosphorylation is insulin dependent, since insulin administration after short-term insulin deficiency reduced tau hyperphosphorylation at selective sites [14]. It has also been described that STZ administration may accelerate amyloid pathology in APP mice by increasing total Aβ levels and SP [46]. This effect might be mediated by AGEs/RAGE/NF-κB pathway modulation [47], and enhanced APP processing has also been reported in this and other AD models [34, 48]. Surprisingly, in our hands APP/PS-STZ-treated mice did not display increased CAA or SP deposition in the cortex or the hippocampus, although we did not specifically checked previously referred pathways, and therefore, it is feasible that APP processing or the AGEs/RAGE/NF-κB pathway could be affected. On the other hand, we are aware of the limited amount of CAA in this animal model at these early stages, which is mainly present in leptomeningeal and penetrating vessels, while we usually detect microhemorrhages deep in the parenchyma. To our knowledge, no previous studies have focused on different Aβ species, and in our hands, STZ-induced insulin depletion slightly reduced SP and insoluble Aβ levels, whereas more toxic soluble Aβ species were favored, as previously described in other AD models with metabolic alterations [12]. It is also possible that increased soluble Aβ levels are an intermediate step in the production of compact deposits, and therefore, assessment of SP deposition at later time points may result in increased Aβ deposition, as previously described [47]. Since Aβ accumulation has been related to an imbalance between production and degradation or clearance of the peptide along perivascular spaces [17], it is also feasible that the observed shift between soluble and insoluble Aβ levels could be due to alterations in any step of the production-elimination process. Following this idea, we analyzed IDE and neprilysin levels in our animals, since Aβ and insulin degrading pathways tend to be reduced with age in AD patients. Previous studies have revealed reduced levels and activities of Aβ and insulin degrading enzymes in diabetic rats [49]; however, our studies are in accordance with previous work on 5xFAD mice in which IDE was not affected after STZ administration [48], limiting a mechanistic approach in our study.

In order to explore other possibilities interfering in Aβ elimination, we assessed the inflammatory process by analyzing microglial activation. We detected an overall increase of microglial burden in APP/PS1 and STZ mice and similar outcomes have been observed after STZ administration to other animal models [50]. Also, this effect was more severe in APP/PS1-STZ mice. We cannot exclude that earlier assessment of the inflammatory process could have shown greater differences, although mice ages and experimental times were selected depending on Aβ pathology, STZ survival, and required time for T1D to fully develop. We also observed that vascular damage, induced by STZ administration, was exacerbated in APP/PS1-STZ-treated mice and hemorrhages were significantly increased in AD-diabetic mice. Whereas the reductions observed in endothelial cells and pericytes are limited, it remains possible that both added alterations may be responsible, at least in part, for observed vascular disease. Vascular alterations have been previously described [50], although to our knowledge, the presence of spontaneous bleeding has not been assessed in this AD-diabetes model, and our results suggest a synergistic effect between both pathologies. These data are in accordance with epidemiological studies showing that vascular disease is also a risk factor for dementia and that long-term hyperglycemia is associated with microvascular complications [51]. Following this idea, it has been also shown that cerebrovascular disease may worsen AD clinical symptoms [52, 53], and it remains possible that blood-brain barrier alterations may be responsible for the observed accumulation of Aβ soluble species and learning and memory dysfunction.

## Conclusions

Altogether, our data suggest that APP/PS1-STZ-treated mice present central alterations including exacerbated inflammation and spontaneous bleeding. These independent and additive adverse effects of STZ and APP/PS1 occurred with respect to AD pathology and may underlie observed cognitive dysfunction. Therefore, it remains possible that by controlling metabolic alterations, as previously suggested [19], central pathological features could also be reduced.

## Material and Methods

### Animals

We used APPswe/PS1dE9 mice (Jackson Laboratory, Bar Harbor, USA) as model of Alzheimer’s disease in this study. In order to induce diabetes, mice were treated as previously described [11]. Mice (18 weeks old) were injected ip with STZ (40 mg/kg) for five consecutive days and animals were aged up to 26 weeks of age without insulin therapy. All experimental procedures were approved by the Animal Care and Use Committee of the University of Cadiz, in accordance with the Guidelines for Care and Use of Experimental Animals (European Commission Directive 2010/63/UE and Spanish Royal Decree RD 53/2013).

### Metabolic Determinations

Body weight, postprandial blood glucose, and insulin levels were determined at 18 (before the commencement of the treatment) and 26 weeks of age as previously described [11]. Briefly, blood glucose levels were measured from nicked tails using the glucometer Optium Xceed (Abbott, UK). Blood for plasma insulin determination was collected from the tail vein into capillary tubes precoated with potassium-EDTA (Sarstedt, Nümbrecht, Germany). Blood samples were centrifuged during 10 min, 6500 rpm at 4 °C, and plasma fraction was stored at −80 °C until processed. Plasma insulin levels were measured using ultrasensitive mouse enzyme-linked immunosorbent assay (ALPCO Diagnostics, Salem, NH, USA).

### MWM

Fourteen days prior to sacrifice, learning and memory abilities were analyzed in the MWM test as previously described [11], with minor modifications. Briefly, the maze consisted of a round tank of water (0.95 m in diameter) with four equal virtual quadrants indicated with geometric cues mounted on the walls. An escape platform was located 2–3 cm below the water surface and camouflaged with calcium carbonate to cloud the water. Water temperature was 21 ± 1 °C. A camera was mounted above the maze and attached to a computer and Smart software (Panlab, Spain). Testing was conducted in two phases: acquisition and retention. Acquisition consisted of four trials per day for 4 days with the platform submerged. During this phase, the platform was located in quadrant 2. The time limit was 60 s/trial with an intertrial interval of 10 min. If the animal did not find the platform, it was placed on it for 10 s. The retention phase took place 24 h (retention 1) and 72 h (retention 2) after finishing the acquisition phase. In this phase, the submerged platform was removed, mice were allowed to swim for 60s, and the time that mice spent in the quadrant where the platform was previously located (quadrant 2) was recorded using SMART system (Panlab, Spain). Swimming velocity was also measured in order to detect any motor activity dysfunction that could bias the learning and memory assessment.

### Motor Function and NOD Task

One day after finishing the MWM task, locomotor activity was assessed in all animals under study as previously described [54]. We measured the distance travelled by the mice for 30 min in a transparent rectangular box (22-cm long × 44-cm width × 40-cm high) using SMART system (Panlab, Spain). In order to further assess anxiety-like behavior in our mice, distance travelled was analyzed in the proximity of the walls, as well as in the center of the boxes (10 cm from the border). One day after actimetry, mice continued with the NOD test to assess their episodic memory as previously described [55]. On day 2 animals were exposed to two objects, for habituation purposes, not used again during the object exploration task on day 3. On day 3 each mouse received two sample trials and a test trial. On the first sample trial, mice were placed into the center of the box containing three copies of a novel object (blue balls) arranged in a triangle-shaped spatial configuration and allowed to explore them for 5 min. After a delay of 30 min, the mice received a second sample trial with four novel objects (red cones), arranged in a quadratic-shaped spatial configuration, for 5 min. After a delay of 30 min, the mice received a test trial with two copies of the object from sample trial 2 (recent objects) placed in the same position and two copies of the object from sample trial 1 (familiar objects): one of them in the same position (familiar non-displaced object) and the other one in a new position (familiar displaced object). Integrated episodic memory for “what,” “where,” and “when” was analyzed as previously described [55]: “What” was defined as the difference in time exploring familiar and recent objects, “where” was defined as the difference in time exploring displaced and non-displaced objects, and “when” was defined as the difference between time exploring familiar non-displaced and recent non-displaced objects. Motor function was also analyzed in the rotarod (Panlab, Spain), as previously described [56], with minor modifications. Briefly, mice were placed in the rotarod facing away the experimenter. The rod accelerated from 0 to 30 rpm over 3 min and final revolutions per minute for each mice were recorded.

### Tissue Processing

At the end of the NOD test, mice were sacrificed by chloral hydrate overdose (60 mg/kg). Brains were rapidly extracted and weighed. Subsequently, hemispheres were separated: The right hemisphere was dissected into the cortex and hippocampus, which were preserved at −80 °C until used, and the left hemisphere was immersed in 4 % paraformaldehyde for 2 weeks at 4 °C, to be then cut into 30-μm coronal sections, for histochemical and immunohistochemical determinations.

### Cresyl Violet Staining

We analyzed brain morphology by cresyl violet staining in sections selected 1 mm apart (from 1.5 to −3.5 mm from bregma) [57]. All six sections were used to analyze cortex morphology and the last three sections were also used for hippocampal studies. Briefly, sections were mounted and dehydrated in 70 % ethanol for 15 min before incubation in cresyl violet (Sigma, St. Louis, MO, USA) solution 0.5 % (*w*/*v*) for 5 min. Sections were washed and fixed in 0.25 % acetic acid in ethanol for 7 min and subsequent 100 % ethanol and xylene for 2 min. Sections were mounted with DPX (Sigma, St. Louis, MO, USA) photographed with a Laser Olympus U-RFLT fluorescent microscope (Olympus, Japan). Images were acquired using MMIcellTools software. Cortex and hippocampus sizes were measured using ImageJ software.

### Prussian Blue Staining

Postmortem study of hemorrhages was performed by Prussian blue iron staining and neutral red counterstain, as previously described with minor modifications [11]. Briefly, adjacent sections to the ones used for cresyl violet staining were mounted on slides and they were exposed to Prussian blue staining (HCl 10 % and potassium ferrocyanide 5 %) for 30 min. Then, they were washed generously with water and rehydrated with 1-M phosphate buffer for 5 min. Subsequently, sections were dehydrated in 70 % alcohol and immersed in a neutral red solution 1 % (*v*/*w*) for 5 min. After washing, sections were fixed in 95 % ethanol (with 1 % acetic acid) and immersed in xylol for 4 min. Sections were mounted with DPX (Sigma, St. Louis, MO, USA), coverslipped and photographed with an Olympus U-RFL Laser-U fluorescent microscope (Olympus, Japan). Cortical and hippocampal images were analyzed with the free software ImageJ.

### Aβ ELISA Measurements

Soluble and insoluble Aβ40 and Aβ42 were quantified in the cortex and hippocampus from groups under study using colorimetric ELISA kits (Wako, Japan, Aβ40 ref 294-62501 and Aβ42 ref 290-62601) as previously described, with minor modifications [20]. At each step, homogenization of 5–10 mg of tissue in 50 ml of lysis buffer with protease inhibitor cocktail (ref: 87787 and 87786, respectively, Thermo Scientific Pierce, Spain) was followed by centrifugation at 14,500 rpm for 12 min at 4 °C. Supernatants were retained for soluble Aβ40 and 42 levels. The resultant pellet was then extracted with 50 ml of 70 % formic acid in distilled water and then centrifuged 10 min at 14,500 rpm at 4 °C, and this fraction was neutralized with 1 M Tris (pH 11). Soluble and insoluble fractions were diluted 1:10 and 1:30, respectively. Standard curves were made using human Aβ40 and Aβ42 standards provided in the ELISA kit. Absorbance was measured spectrophotometrically at 450 nm (MQX200R2, Biotek instruments, Burlington VT, USA) and data were expressed as picomole per gram wet tissue.

### Amyloid-Beta, Microglia, Endothelium, Pericytes, and Neuronal Immunohistochemistry

APP/PS1-STZ and APP/PS1-Sham mice were assessed postmortem for Aβ burden in the cortex and hippocampus. Immunohistochemistry for Aβ was performed as previously described [12] with minor modifications. PFA-fixed 30-μm sections were washed in PBS and pretreated with 70 % formic acid for 10 min, and subsequently, they were blocked in 5 % normal goat serum (NGS) and 0.5 % Triton-X 100 during 1 h. Sections were immunostained with anti-Aβ17-24 antibody 1:2000 (4G8, Covance, Spain) and anti-IBA1 1:1000 (Wako, Japan) in 1 % NGS overnight at 4 °C, followed by secondary goat anti-rabbit conjugated to Alexa 488 and goat anti-mouse to Alexa 595 (Invitrogen, USA) in 1 % NGS 1 h at room temperature. Images were acquired using a Laser Olympus U-RFL-T fluorescent microscope (Olympus, Japan) and MMIcellTools software and analyzed with ImageJ software to quantify Aβ burden as well as the number and size of individual SP. CAA was also quantified as previously described [20] and results were expressed as CAA burden (% of affected vessel). Endothelium (anti-CD31, 1:100, BD Bioscience, USA ) and pericyte (anti-NG2, 1:100, Millipore, USA) immunostaining was performed in the same conditions. Alexa Fluor anti-rat 594 and Alexa Fluor anti-rabbit 488 (Life Technologies, USA) were used as secondary antibodies, respectively, and percentage of vessel area covered by endothelial cells or pericytes was quantified with ImageJ software. Microglial activation was measured in the close proximity of plaques (up to 50 μm from SP) as well as far from SP (>50 μm), in the case of APP/PS1-Sham and APP/PS1-STZ-treated mice. In the case of wild-type mice, the same parameters were measured and compared with microglia far from plaques in transgenic mice. Sections selected 1 mm apart (from 1.5 to −3.5 mm from bregma), in all animals under study, were coded and blind quantification was manually performed in the regions of interest (cortex or hippocampus) using ImageJ software. To quantify microglial burden (% area covered), number of cells, and individual microglial size, the final analysis included between 4500–6900 cells/group in the cortex and 250–614 cells/group in the hippocampus. Contiguous sections that were used for neuronal immunostaining were incubated in anti-NeuN antibody (Chemicon) 1:200, and conjugated goat anti-mouse Alexa 594 was used as secondary antibody. Sections were washed and stained with 4′,6-diamidino-2-phenylindole (DAPI) 1 mg/ml (Sigma) (1:2000) and thioflavin S 0.001 %. The percentage of NeuN-positive cells (normalized by total cells stained with DAPI) was quantified in the proximity of SP in APP/PS1 and APP/PS1-STZ mice (up to 50 μm). Areas located far from SP were also compared with wild type and wild-type-STZ mice using Image J software.

### IDE, Neprilysin, Total Tau, and Phospho-tau Levels

Western blot for IDE, NEP, phospho-tau, and total-tau levels was performed in fresh tissue as previously described [12]. Briefly, 5–10 mg of tissue from the cortex and hippocampus was pretreated in lysis buffer (Cell Signaling, USA) containing protease inhibitors and phosphatase inhibitors (Sigma, USA). Protein (80 μg) in the case of total and phospho-tau, and 160 μg in the case of IDE and NEP, was loaded and separated on 10 % acrylamide-bisacrylamide gels. Proteins were transferred to PVDF membranes. Membranes were then immersed in blocking buffer (Invitrogen) for 1 h and incubated overnight at 4 °C with primary antibodies anti-total tau (1:1000; DAKO, Glostrup, Denmark), anti-phospho-tau (1:1000, clone AT8; Fisher Scientific, Waltham, MA, USA), anti-IDE (1:1000, N-Terminal 97-273, PC730, Millipore), anti-NEP (1:1000, ab951, Abcam), and anti-β-actin (1:2,500,000, Sigma). Membranes were washed and then incubated with a chemiluminescent immunodetection system for primary antibodies for mouse (AT8, anti-IDE, and anti-β-actin) or for rabbit (anti-total tau and anti-NEP) (Invitrogen, Carlsbad, USA) for 1 h. Signal was detected using Novex AP Chemiluminescent Substrate (Invitrogen, Carlsbad, USA) and Kodak Biomax Light Film (Sigma, USA). Subsequent primary antibody incubations were preceded by stripping, using Western Blot Stripping Buffer (Fisher Scientific, Waltham, MA, USA) for 10 min at 37 °C in agitation. Immunoblots were semi-quantified by measuring the optical density (OD) of each protein band on scanned film using the ImageJ software. Data were represented as percentage of wild-type-Sham values.

### Statistical Analysis

Two-way ANOVA was performed to compare the acquisition phase in the MWM test. Further differences in the MWM and the NOD tests, as well as in postmortem studies (histology, immunohistochemistry, and Western blot), were determined by one-way ANOVA followed by Tukey *b* or Tamhane tests as required. When only two groups (APP/PS1-Sham and APP/PS1-STZ) were under study, differences were detected by Student’s *t* test for independent samples, whereas one-way ANOVA was used when more than two groups were under study. SPSS v.15 software package was used for all statistical analysis.
